# Prehospital emergency medical service utilization and associated factors among critically ill COVID-19 patients treated at centers in Addis Ababa, Ethiopia

**DOI:** 10.1371/journal.pgph.0001158

**Published:** 2023-02-01

**Authors:** Ararso Baru Olani, Lemlem Beza, Menbeu Sultan, Tariku Bekelcho, Michael Alemayehu

**Affiliations:** 1 College of Medicine and Health Sciences, Arbaminch University, Arbaminch, Ethiopia; 2 Department of Emergency Medicine and Critical Care, Addis Ababa University, Addis Ababa, Ethiopia; 3 Department of Emergency Medicine and Critical Care, Saint Paul’s Hospital Millennium Medical College, Addis Ababa, Ethiopia; 4 Tirunesh Beijing Hospital, Addis Ababa, Ethiopia; University of Embu, KENYA

## Abstract

The majority of populations in developing countries are living in areas of no access or limited access to prehospital emergency medical services (EMS). In Addis Ababa, the reported prehospital EMS utilization were ranging from zero to thirty-eight percent. However, there is limited research on reasons for the low utilization of prehospital resources in Ethiopia. This study aimed to assess factors associated with prehospital EMS utilization among critically ill COVID-19 patients in Addis Ababa, Ethiopia. A hospital-based cross-sectional study was conducted to collect primary data from 421 COVID-19 patients in Addis Ababa between May and July 2021. Logistic regression was used to identify factors associated with prehospital service utilization. Andersen’s Behavioral Model was implemented to address independent variables, including predisposing, enabling, need, and health behaviors-related variables. The level of prehospital care utilization was 87.6%. Being married [AOR 2.6(95%; CI:1.24–5.58)], belief that self-transport is quicker than the ambulance [AOR 0.13(95%; CI: 0.05–0.34)], and perceptions that ambulance provides transportation service only [AOR 0.14(95%; CI:0.04–0.45)] were predisposing factors associated with prehospital service utilization while the source of referrals [AOR 6.9(95%; CI: 2.78–17.30)], and prior knowledge on the availability of toll-free ambulance calling numbers [AOR 0.14(95%; CI: 0.04–0.45)] were identified as enabling factors. Substantial proportions of critically ill COVID-19 patients used prehospital services to access treatment centers. Prehospital EMS utilization in this study varies by predisposing and enabling factors, particularly: marital status, source of referral, prior knowledge on the availability of toll-free ambulances, belief that self-transport is quicker than ambulances, and perceptions that ambulance provides transportation service only. Our findings call for further actions to be taken by policymakers including physical and media campaigns focusing on the identified factors.

## Introduction

There is a growing need for prehospital emergency services worldwide as a result of the global increase in the emerging and evolving burden of diseases that require emergency care systems for prehospital treatment and/or transport to a hospital for further treatment [1–4]. Similarly, critical care demand in emergency departments is disproportionately increasing due to traumatic and non-traumatic illnesses such as road traffic collisions and COVID-19 [5–7].

Severe infection with COVID-19 can induce a life-threatening condition such as acute respiratory distress syndrome (ARDS) [8–10], which needs urgent transfer to specialized centers for escalating treatment [11, 12]. Transport by EMS, as opposed to self-transport, is associated with better patient outcomes by providing access to prehospital lifesaving interventions and rapid transport to appropriate facilities [13–15]. However, the majority of the population in low-income countries are living in areas with no access or limited access to formal emergency care systems [16]. Only less than 1% of the population in many low-income countries have access to transportation by ambulances [17]. Similar to other low-income countries, only few patients are presenting by ambulance when experiencing traumatic and non-traumatic emergency medical conditions in Ethiopia [18–20]. Studies reported that the proportion of patients who received prehospital care for emergency illness or injury in Ethiopia were ranging from zero [19, 20] to thirty-eight percent [21].

As of January 26, 2022, a total of 463,047 confirmed cases and 7,280 deaths from COVID-19 were reported to the World Health Organization (WHO) from Ethiopia [22]. As a national response to tackle the COVID-19 pandemic, Addis Ababa City Health Burau and the Ethiopian Federal Ministry of Health have established charge-free EMS for COVID-19 patients. However, to our knowledge, there is a dearth of literature showing prehospital EMS utilization among COVID-19 patients in the city. In addition, little is known about factors associated with prehospital services in general and COVID-19 patients in particular during an emergency in Ethiopia. Therefore, this study aimed to assess the magnitude and factors associated with prehospital emergency medical service utilization among COVID-19 patients treated at treatment centers in Addis Ababa, Ethiopia.

## Methods and materials

### Study setting and period

The study was carried out in Addis Ababa city, the capital city of Ethiopia, and the seat of the Africa Union. The city is served by public and private ambulances. Fourteen public health facilities provide treatment to COVID-19 patients. The study was conducted between May and July 2021 at four centers that provide dedicated services to COVID-19 patients namely: Eka Kobe Hospital (EKH), Millennium Hall COVID-19 treatment center (MHCTC), Saint Paul’s Hospital (SPH), and Field Hospital (FH).

### Study design

An institutional-based cross-sectional study was carried out to assess factors associated with prehospital service utilization among critically ill COVID-19 patients treated at centers in Addis Ababa, Ethiopia.

### Eligibility criteria

The study included all patients aged 18 years and above, diagnosed with severe or critical COVID-19, and who volunteer to participate in the study. On the other hand, mild to moderate COVID-19 patients, those unconscious on arrival, and who refused to provide consent were excluded from the study.

### Sample size determination

A single population proportion formula was used to calculate the sample size. Given that there was no previous study conducted on the mode of arrival to hospital among COVID-19 patients in Ethiopia, a 50% prevalence of ambulance use was taken. Considering a 5% margin of error at a 95% confidence level, and adding 10% for non-response rate a total of 422 samples from selected hospitals were recruited.

### Data collection tool and quality assurance

Data collection was done using an interviewer-administered structured questionnaire. The data collection tool was prepared in English, which was developed following reviews of previous literature. The tool consisted of both open and close-ended questions, which included sociodemographic characteristics, prehospital care-related characteristics, and perceived and observed clinical characteristics of the patients.

The training was provided to data collectors and supervisors regarding the tool ahead of data collection. The validity, practicability, and interpretability of responses for each question on the tool were confirmed by conducting a pretest on 5% of the sample sizes (21 respondents). In addition, based on the feedback from the pre-test study, the format and wording of questions were corrected and refined.

### Measurements

#### Outcome variables

The outcome variable, which was prehospital care utilization, was measured by asking whether the patient arrived at the hospital by ambulance and received any intervention from the ambulance crew during the transportation or not. If the respondent arrived hospital by ambulance and received some kind of intervention during transport we coded it as 1, otherwise, we coded it as 0.

#### Covariates

Andersen’s Behavioral Model (ABM) was implemented to conceptualize and measure covariates. ABM is the most widely used conceptual model for explaining variation in the use of health services [23]. According to ABM health care service utilization is determined by predisposing factors, enabling factors, need factors, and health behaviors [24]. Prehospital EMS use could be determined by predisposing factors such as age, sex, educational status, marital status, living arrangement, the language the patient speaks, attitude toward the use of emergency medical services; enabling factors including time of the day, date of presentation to hospital, awareness of the existence of charge-free ambulance services, accessibility of the services, possession of mobile phones, and waiting time for the ambulance to arrive; need factors like perceived health status, perceived severity of the disease, and comorbid medical conditions; health behaviors such as previous use of EMS.

### Data analysis

The data were evaluated for completeness, cleaned, coded, and entered into Epidata version 3.1 for validation. SPSS version 25 was used for the analysis of the data. Descriptive analysis was performed to summarize the findings while tables and diagrams were used to present the information. Logistic regression was conducted to test associations between dependent and independent variables. To control the effect of confounders, all variables with a p-value cut-off point of < 0.25 on bivariate analysis were purposively selected for multivariable logistic regression analysis. These variables were: educational status, marital status, ability to speak Amharic, living arrangement, source of referral, day of presentation, chronic illness, Diabetic Mellitus, high blood pressure, awareness of toll-free ambulance service for COVID-19 patients, awareness of charge-free ambulance services for COVID-19 patients, belief that patients transported by ambulance receive quality care with compassion, belief that self-transport is quicker than an ambulance, and belief that ambulance crew provides transport service only. A statistically significant association was declared at a cut-off p-value of p<0.05.

### Ethics statement

Ethical approval was obtained from the institutional review board (IRB) of Eka Kotebe General hospital with IRB number EKGH/10/5/85. An official letter of cooperation was also written from the Federal Ministry of Health, and Emergency Directorate to respective institutions, and permission to conduct the study was received from each provost of the selected institutions. In addition, informed verbal consent was secured from each study participant. Personal unique identifiers such as the name of the study participants were not taken. The confidentiality of the information collected from the respondents was maintained. Furthermore, all procedures involved in this study have adhered to the principles of the Helsinki Declaration.

### Operational definitions

The operational definitions for COVID-19 severity levels were adopted from the national COVID-19 management guideline of the Federal Democratic Republic of Ethiopia [25].

Severe COVID-19: was described as a COVID-19 patient having severe pneumonia, ARDS, sepsis, or patients responding to non-invasive management. These patients manifest with dyspnea, respiratory rate ≥ 30/min, blood oxygen saturation (SpO2) ≤ 90%, or when there is arterial blood gas PaO2/FiO2 ratio < 300 or when Kigali definition is used SpO2/FIO2<350, and/or lung infiltrates in computed tomography imaging > 50% within 24 to 48 hours.

Critical COVID-19: was described as a COVID-19-positive patient with respiratory failure, septic shock, and/or multiple organ dysfunctions or failure and needs invasive or special management.

## Results

A total of 421 samples were included in the analysis, giving a response rate of 99.8%. One sample was excluded from the analysis because of incomplete data following the withdrawal of the patient from the study.

### Socio-demographic characteristics (predisposing characteristics) of the respondents

The mean age of severe and critical COVID-19 patients included in this study was 51.6 years with a standard deviation (SD) of 18.1. Over one-fourths 118(28.0%) of the participants were in the age group of ≥ 65 years and the majority 244(58.0%) of the respondents were male (Table 1).

**Table 1:** Description of socio-demographic (predisposing) characteristics by prehospital care utilization among severe and critical COVID-19 patients, Addis Ababa, Ethiopia, May to July 2021.

Variables	Frequency (percentage)	Received prehospital care		p-value (χ2 test)
		Yes	No	
**Age in years (Mean = 51.6 years; SD = 18.1)**				0.527
<25	40(9.5)	33	7	
25–34	54(12.8)	46	8	
35–44	52(12.4)	46	6	
45–54	82(19.5)	73	9	
55–64	75(17.8)	70	5	
≥65	118(28.0)	101	17	
**Sex**				0.521
Male	244(58.0)	216	28	
Female	177(42.0)	153	24	
**The highest level of education attended**				0.122
Can’t read and write	60(14.3)	54	6	
Primary school	148(35.2)	122	26	
Secondary school	91(21.6)	80	11	
Diploma or vocational school	43(10.2)	41	2	
Bachelor’s degree and above	79(18.8)	72	7	
**Marital status**				**0.004**
Married	270(64.1)	246	24	
Unmarried	151(35.9)	123	28	
**Patient able to speak Amharic**				0.196
Yes	371(88.1)	328	43	
No	50(11.9)	41	9	
**Living arrangement**				0.179
**Alone**	84(20.0)	70	14	
**Not alone**	337(80.0)	299	38	

### Enabling characteristics of the respondents

The majority of 388(92.2%) study participants were referred from healthcare facilities. Over two-thirds of 299(71.0%) patients arrived at treatment centers during the day time and the majority 356(84.6) of them arrived at the hospitals during the weekday compared to weekends (Table 2).

**Table 2:** Description of enabling characteristics by ambulance utilization among severe and critical COVID-19 patients who visited treatment centers in Addis Ababa, Ethiopia, from May to July 2021.

Variables	Frequency (percentage)	Received prehospital care		p-value (χ2 test)
		Yes	No	
**Source of referral**				**0.001**
Self-referral	33(7.8)	20	13	
Public health care facility	388(92.2)	349	39	
**Day of presentation**				0.104
Weekday	356(84.6)	316	40	
Weekend	65(15.4)	53	12	
**Time at presentation**				0.982
Day	299(71.0)	262	37	
Night	122 (29.0)	107	15	
**Possessed mobile phone**				0.554
Yes	285(67.7)	249	36	
No	132(31.4)	118	14	
**Aware availability of toll-free ambulance services to transport COVID-19 patients**				**0.001**
Yes	382(90.7)	348	34	
No	39(9.3)	21	18	
**Aware availability of charge-free ambulance services to transport COVID-19 patients**				**0.001**
Yes	373(88.6)	337	36	
No	48(11.4)	32	16	

### Respondents’ beliefs on prehospital care (predisposing characteristics)

Most (92.2%) participants believed that patients transported by ambulance receive quality care with compassion and only 29(6.9%) responded that non-ambulance vehicles were a faster transport mode in reaching hospital (Table 3).

**Table 3:** Description of participant beliefs (predisposing characteristics) by prehospital care utilization among severe and critical COVID-19 patients who visited treatment centers in Addis Ababa, Ethiopia, from May to July 2021.

Variables	Frequency (percentage)	Received prehospital care		p-value (χ2 test)
		Yes	No	
**I believe that patients transported by ambulance receive quality care with compassion**				0.107
Agree	388(92.2)	343	45	
Disagree	33(7.8)	26	7	
**I believe that self-transport is quicker than an ambulance**				**0.001**
Agree	29(6.9)	18	11	
Disagree	392(93.1)	351	41	
**I believe that the use of non-ambulance transport is safer than an ambulance**				0.840
Agree	27(6.4)	24	3	
Disagree	394(93.6)	345	49	
**I perceive that the ambulance crew provides only transport services**				**0.014**
Agree	188(44.7)	173	15	
Disagree	233(55.3)	196	37	

### Need characteristics of the respondents

The majority of 246(58.4%) study participants had a Glasgow coma scale (GCS) score of ≥13. A total of 241(57.2%) respondents reported having a history of at least one chronic medical illness. Of which, diabetes mellitus was the commonest 119(28.3%) followed by hypertension 116(27.6%) (Table 4).

**Table 4:** Description of prehospital care utilization by need factors among severe and critical COVID-19 patients who visited treatment centers in Addis Ababa, Ethiopia, from May to July 2021.

Variables	Frequency (percentage)	Received prehospital care		p-value (χ2 test)
		Yes	No	
**Perceived physical health status**				0.298
Excellent	71(16.9)	61	10	
Very good	99(23.5)	88	11	
Good	150(35.6)	133	17	
Fair	55(13.1)	44	11	
Poor	46(10.9)	43	3	
**Mental status (evaluated by GCS)**				0.374
≤8	37(8.8)	30	7	
9–12	138(32.8)	120	18	
≥13	246(58.4)	219	27	
**At least one comorbid chronic illness**				**0.020**
Yes	241(57.2)	219	22	
No	180(42.8)	150	30	
**Diabetes mellitus**				**0.028**
Yes	119(28.3)	111	8	
No	302(71.7)	258	44	
**Hypertensions**				**0.036**
Yes	116(27.6)	108	8	
No	305(72.4)	261	44	
**Chronic heart diseases**				0.596
Yes	55(13.1)	47	8	
No	366(86.9)	322	44	
**Chronic kidney diseases**				0.979
Yes	32(7.6)	28	4	
No	389(92.4)	341	48	
**Chronic respiratory diseases**				0.253
Yes	22(5.2)	21	1	
No	399(94.8)	348	51	

### Health behavior of the respondents and their family

Most 287(68.2%) of the study participants had no previous experience of transport by ambulance for non-COVID-19 related emergencies and only 62(14.7%) of them reported having family member/s previously transported by ambulance for COVID-19-related emergencies (Table 5).

**Table 5:** Description of health behaviors of the patients and their families by prehospital care utilization among severe and critical COVID-19 patients treated in centers in Addis Ababa, Ethiopia, from May to July 2021.

Variables	Frequency (percentage)	Received prehospital care		p-value (χ2 test)
		Yes	No	
**The patient used an ambulance for non-COVID-9-related emergencies in their lifetime**				0.622
Yes	134(31.8)	119	15	
No	287(68.2)	250	37	
**The patient’s family member/s previously used an ambulance for COVID-19-related emergencies.**				0.575
Yes	62(14.7)	53	9	
No	359(85.3)	316	43	

### Level of prehospital emergency medical service utilization

The level of prehospital emergency medical service utilization among COVID-19 patients in this study was identified as 87.6%.

### Logistic regression of factors associated with prehospital care utilization

Factors that were associated with prehospital care utilization are summarized and presented in Table 6. Of the socio-demographic predisposing variables, marital status showed statistically significant associations with prehospital care utilization among COVID-19 patients after adjusting for potential confounders [AOR 2.6(95%; CI:1.24–5.58)]. Implying that, married COVID-19 patients were 2.6 times more likely to utilize prehospital care services compared to unmarried COVID-19 patients. We also found that the COVID-19 patients who thought self-transport would get them to hospital faster than ambulances were 87% less likely to utilize prehospital services compared to their counterparts, [AOR 0.13(95%; CI: 0.05–0.34)]. Patient’s perception of the role of the ambulance crew was another predisposing factor associated with prehospital care service utilization, [AOR 0.14(95%; CI:0.04–0.45)]. Indicating that, the respondent who thought ambulance crew provides only transport services were 86% less likely to use EMS for COVID-19 compared to their counterparts.

**Table 6:** Bivariate and multivariate logistic regression analysis of factors associated with prehospital care utilization among severe and critical COVID-19 patients treated in centers in Addis Ababa, Ethiopia, from May to July 2021.

Variables	Used PEMS		COR (95%) CI	AOR (95%) CI
	Yes	No		
**Predisposing factors**				
**Marital status**				
Married	246	24	2.3(1.30–4.19) **	2.6(1.24–5.58) *
Unmarried	123	28	Ref.	Ref.
**I believe that self-transport is quicker than the ambulance**				
Agree	18	11	0.19(0.08–0.43) ***	0.13(0.05–0.34) ***
Disagree	351	41	Ref.	Ref.
**I perceive that the ambulance crew provides only transportation to the patient**				
Agree	348	34	Ref.	Ref.
Disagree	21	18	0.11(0.06–0.23) ***	0.14(0.04–0.45) **
**Enabling factors**				
**Source of referral**				
Self-referral	20	13	Ref.	Ref.
Public health care facility	349	39	5.8(2.67–12.60) ***	6.9(2.78–17.30) ***
**Aware availability of toll-free ambulance services to transport COVID-19 patients**				
Yes	348	34	Ref.	Ref.
No	21	18	0.11(0.06–0.23) ***	0.14(0.04–0.45) **
**Aware availability of charge-free ambulance services to transport COVID-19 patients**				
Yes	337	36	Ref.	Ref.
No	32	16	0.21(0.11–0.43) ***	0.65(0.20–2.13)
**Need factors**				
**At least one comorbid chronic illness**				
Yes	219	22	2.0(1.11–3.58) *	1.7(0.70–4.38)
No	150	30	Ref.	Ref.
**Diabetes mellitus**				
Yes	111	8	2.4(1.08–5.19) *	1.3(0.52–3.41)
No	258	44	Ref.	Ref.
**Hypertension**				
Yes	108	8	2.3(1.04–4.99) *	1.5(0.53–4.30)
No	261	44	Ref.	Ref.

*p≤0.05

**p≤0.01

***p≤0.001;

PEMS: prehospital emergency medical services; COR: crude odds ratio; AOR; Adjusted odds ratio

Of the enabling variables, the source of referral was independently associated with prehospital care utilization [AOR 6.9(95%; CI: 2.78–17.30)]. Implying that, the patients referred from public health care facilities were 6.9 times more likely to use prehospital EMS compared to self-referral patients. In addition, the patients who had no awareness of the availability of toll-free ambulance calling numbers were 86% times less likely to use prehospital care services compared to others [AOR 0.14(95%; CI: 0.04–0.45)] (Table 6).

A statistically significant association was not observed for need factors on multivariate logistic regression. However, having at least one chronic comorbid illness [COR 2.0(95%; CI: 1.11–3.58)]; known diabetes [COR 2.4(95%; CI;1.08–5.19)] and a history of hypertension [COR 2.3(95%; CI: 1.04–4.99)] were associated with prehospital care service utilization without adjusting for potential confounders (Table 6).

## Discussion

The importance of prehospital care use is centered on its capacity to increase the chance of survival and reduce morbidity related to emergency medical illness [26, 27]. However, these life-saving opportunities can be missed because of a lack of access to the service [27]. We identified that 87.6% of COVID-19 patients included in this study utilized prehospital emergency medical services. Our results were far higher than that of previous studies conducted in the same city, before and during the COVID-19 outbreak, among non-COVID-19 patients [19–21]. The studies conducted before the COVID-19 outbreak reported levels of prehospital emergency medical service use ranging from zero [19, 20] to 37.7% [21]. Meanwhile, the study conducted during the COVID-19 outbreak in the same city reported a 33% prevalence of prehospital emergency medical service utilization [28]. The possible explanations for the disparity could be that as a national response to the COVID-19 outbreak, Addis Ababa has activated toll-free hotlines and service free of charge prehospital emergency medical services dedicated to transporting COVID-19 patients [29, 30], which could increase the utilization of the service in the present study. Although our finding shows a relatively higher level of prehospital service use among COVID-19 patients, it does not mean the recorded level of emergency medical services utilization is sufficient. Therefore, we suggest more utilization of prehospital services because seriously ill COVID-19 patients are prone to ARDS [9, 10, 31], and the spread of the virus to others could also be higher in the self-transport mode compared to transportation by EMS providers.

Marital status was associated with prehospital care use in this study. Previous studies from Ethiopia and elsewhere seldom reported an association between marital status and prehospital care utilization [27, 32, 33]. Although we could not find literature on the association of marital status with prehospital services utilization, marital status was reported as an independent determinant of ED uses in previous work [34]. The present study identified that COVID-19 patients who thought self-transport would get them to the hospital faster than an ambulance were 87% less likely to use prehospital services compared to their counterparts. The belief in self-transport as a quicker mode of arrival to the hospital was previously evidenced in Australia among coronary artery disease (CAD) patients [35]. A previously reported study conducted in Addis Ababa reported that the average perceived waiting time by the patients attending hospitals for various reasons was 40 minutes, which was beyond the acceptable level [36]. Therefore, our explanation for the present finding could be that people may think waiting for the ambulance to arrive during an emergency may take a long time and give the patient less time to survive.

A previous study from Ethiopia reported that most of the patients that used ambulances were transported from health care facilities than the community [37]. Although we did not assess whether the patients included in this study were transported between facilities or from the community to hospitals, we found that most of them were referred from the health care facilities. Considering the previously reported situation in Addis Ababa [37], we were not surprised by our finding that the patients referred from health care facilities were more likely transported by ambulance than self-referred patients. Therefore, our findings suggest that there is a need to take further actions that make EMS more inclusive and minimize disparities in accessing the services.

The current study shows that prior knowledge of the existence of toll-free numbers to transport COVID-19 patients was independently associated with prehospital emergency medical service use. In agreement with our finding, a previous study from North Ethiopia reported an association between ambulance use and prior knowledge of ambulance calling numbers among laboring women [32].

Although a statistically significant association was not established on adjusted analysis in this study we have observed a significant association between respondents’ awareness of the availability of free ambulance services to transport COVID-19 patients and prehospital services utilization on unadjusted analysis. The study from Mekele city, the capital of Tigray regional state of Ethiopia, evidenced an association between ambulance use and participants’ prior knowledge of the availability of charge-free prehospital services for laboring mothers [32]. Knowledge of toll-free ambulance numbers and availability of charge-free ambulance services or insurances for critically ill patients maximized utilization of prehospital care services in several countries.

### Limitations

One of the limitations of this study is that the respondents were recruited only from public COVID-19 treatment centers. Patients treated at private COVID-19 treatment centers were not included in the study and this may limit the generalizability of our findings. Although it could be one potential enabling factor, the variable monthly income was removed from the analysis because more than half of the respondents resisted disclosing their monthly income and this may affect our findings. Lastly, it was a cross-sectional study with the usual inherent limitations. Therefore, the generalization of our findings should be made considering the mentioned limitations.

Despite the aforementioned limitations, this study was conducted in a multicenter, guided by a model to address explanatory variables, and focused on less researched aspects of COVID-19 research (prehospital care).

## Conclusion

We identified that about nine in ten seriously ill COVID-19 patients used prehospital emergency medical services to access treatment centers. EMS use in this study varies by predisposing and enabling factors. The identified predisposing factors were marital status, belief that self-transport is quicker than ambulance, and perception that ambulance crew provides transport service only. Meanwhile, the identified enabling variables were a source of referrals and prior knowledge on the availability of toll-free numbers to call ambulances. Our findings call for further actions to be taken by policymakers including physical and media campaigns addressing the identified factors.

## Supporting information

S1 Data Supporting file. (SAV)

## Abbreviations

ABM: Andersen’s Behavioral Model
ABO: Ararso Baru Olani
AOR: Adjusted odds ratio
ARDS: Acute respiratory distress syndrome
CAD: Coronary artery diseases
CI: Confidence interval
COR: Crude odds ratio
ED: Emergency department
EKH: Eka kotebe hospital
EMS: Emergency medical services
FH: Field hospital
GCS: Glasgow coma scale
LB: Lemlem Beza
MHCTC: Millennium hall COVID-19 treatment center
MS: Menbeu Sultan
PEMS: prehospital emergency medical services
RTA: Road traffic accidents
SD: Standard deviation
SPH: Saint Paul’s hospital
SPSS: Statistical Package for Social Sciences
TB: Tariku Bekelcho
